# Linking phenotype to genotype using comprehensive genomic comparisons

**DOI:** 10.1016/j.gde.2025.102384

**Published:** 2025-07-24

**Authors:** Leon Hilgers, Michael Hiller

**Affiliations:** 1 Senckenberg Research Institute, Senckenberganlage 25, 60325 Frankfurt, Germany; 2 Institute of Cell Biology and Neuroscience, Faculty of Biosciences, Goethe University Frankfurt, Max-von-Laue-Str. 9, 60438 Frankfurt, Germany

## Abstract

Comparative genomics is a powerful approach to illuminate the genetic basis of phenotypic diversity across macro-evolutionary timescales. Recent advances in sequencing, genome assembly, annotation, and comparative methods promoted large-scale analyses that unveiled genomic determinants contributing to differences in cognition, metabolism, and body plans as well as phenotypes with biomedical relevance, such as cancer resistance, longevity, and viral tolerance. These studies highlight joint contributions of multiple molecular mechanisms and indicate an underappreciated role for gene and enhancer losses driving phenotypic change. However, challenges remain, including comprehensive phenotype databases and genome annotations, improved approaches for identifying lineage-specific adaptations, and functional tests. Here, we review recent progress, highlight major discoveries, and discuss future directions for linking phenotype to genotype using comparative genomics.

## Introduction

**Figure 1:**
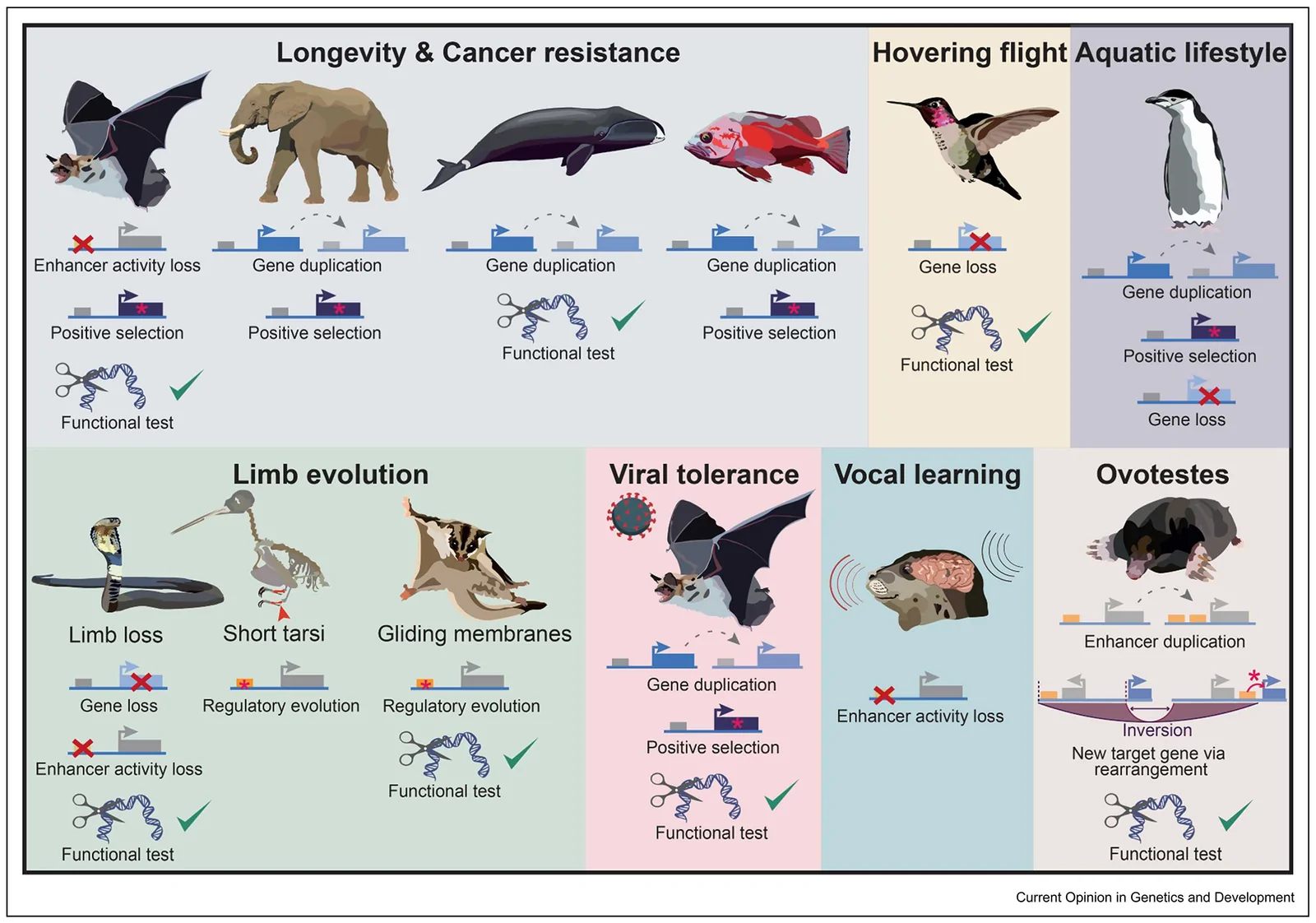
The concept of linking phenotype to genotype using comparative genomics. Comparative genomics uses genome assemblies, genome annotations, and phylogenetic knowledge to reveal genomic differences in functional genomic elements, such as gene losses, signatures of positive selection on protein-coding genes, gene duplications and gene family expansions, and losses or accelerated evolution of CNEs as proxies for *cis*-regulatory elements. The evolutionary history of the phenotype of interest can be inferred from phenotypic data of extant species and the phylogeny. Intersecting data on genomic and phenotypic evolution allow linking phenotypic to genomic differences, which can be assisted by functional annotations of genomic elements. Recapitulating the same or similar genomic differences in model organisms or other experimental systems allows testing whether the uncovered genomic differences contribute to the phenotype of interest.

Understanding the genetic basis of phenotypic differences is a central goal in evolutionary biology. This knowledge is crucial for uncovering phenotypes with translational potential in biotechnology and human health, preserving biodiversity, and predicting evolutionary responses to global change [1]. Until recently, linking phenotypes to genotypes was often limited to model organisms and studying relatively young phenotypic diversity. For example, genetic screens in model organisms and association or quantitative trait loci mapping allowed linking phenotypes to genotypes within a species or across closely related species. By utilizing reference genomes of many different species, phylogenetic data, and natural phenotypic variation, comparative genomics approaches expanded the scope of linking phenotype to genotype to macro-evolutionary timescales (Figure 1).

Here, we review recent developments in genome sequencing and comparative genomics methods, highlight insights into the genomic basis of diverse phenotypes with a focus on vertebrates, and discuss remaining challenges (Box 1).

### Progress in genome sequencing and assembly

**Figure 2:**
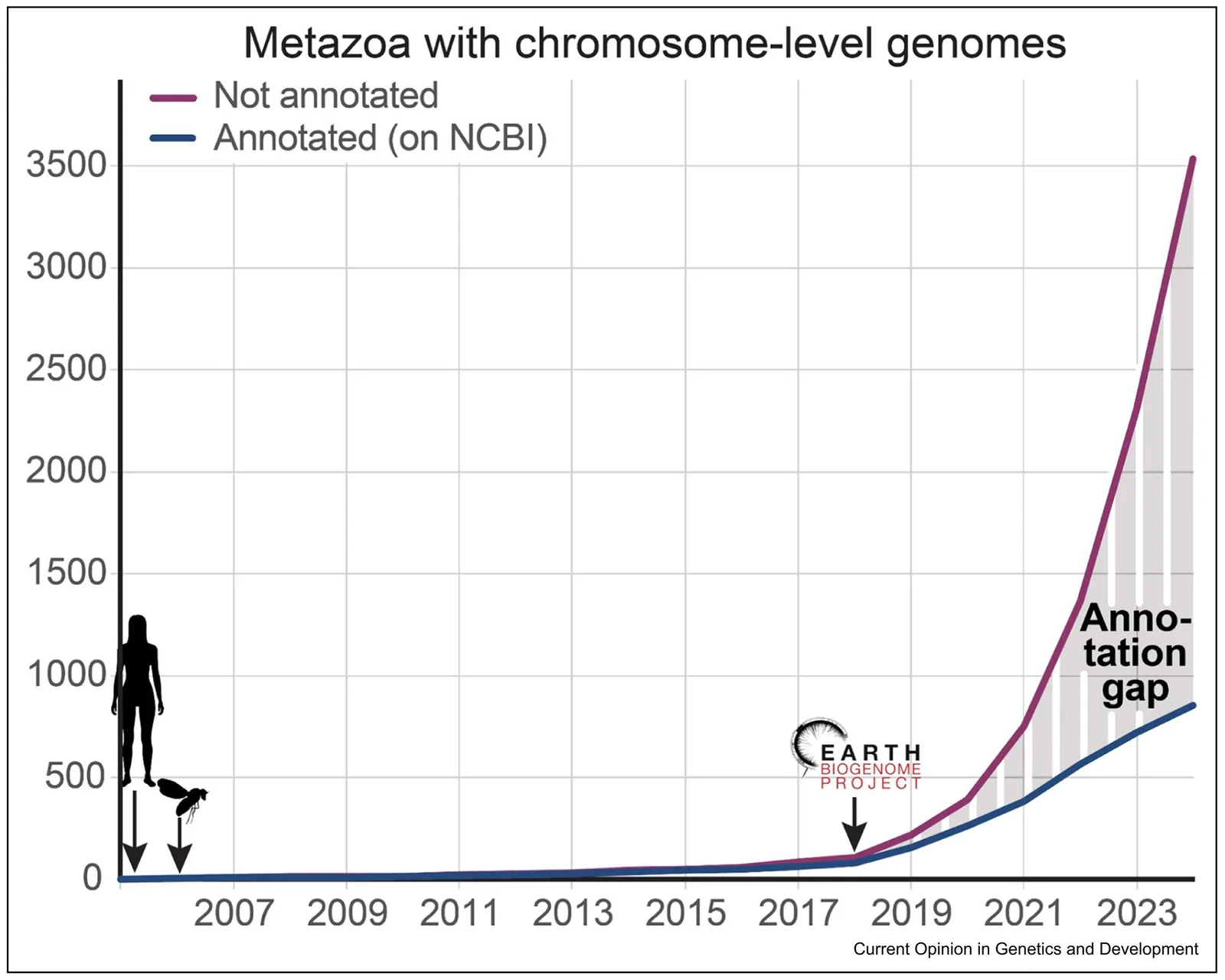
Exponential increase in reference genomes with a growing “annotation gap.” The number of Metazoan taxa with any available chromosome-level genomes (purple) and annotated chromosome-level genomes (blue). Icons indicate the availability of such genomes for the human and *Drosophila*, as well as the start of the Earth Biogenome Project. This figure was generated using data on published genomes and annotations from NCBI (National Center for Biotechnology Information) until January 1, 2025 (https://www.ncbi.nlm.nih.gov/datasets/genome/; accessed on February 6, 2025). Although NCBI is a common source for annotations, we note that annotations provided by community-driven databases or generated by TOGA (Tool to infer Orthologs from Genome Alignments) are not consistently present on NCBI.

Advancements in genome sequencing and assembly avoid previously common artifacts, such as missing sequences, false duplications, and base errors, which hinder accurate comparative genomics analyses [2,3]. Progress in long-read sequencing massively improved assembly contiguity, and cost-efficient scaffolding techniques like chromosome conformation capture (Hi-C) enabled generating highly complete, chromosome-level reference genomes. Even telomere-to-telomere (T2T) genome assemblies that resolve all difficult-to-assemble loci as well as repeat-rich and exceptionally large genomes like the South American Lungfish genome (~30 times human genome size) can now be assembled [4–7]. Although T2T assemblies require increased efforts, which currently limits them to selected species, state-of-the-art methods now routinely produce high-quality, base-accurate, haplotype-resolved assemblies of diploid species using only accurate long-read and Hi-C data [8,9]. Thus, efforts from individual labs and global sequencing consortia have generated thousands of high-quality reference genomes [10–13] (Figure 2), which provide unprecedented opportunities.

### Progress in annotation and orthology inference

Comparing genes across species requires distinguishing between orthologous and paralogous genes that originated from speciation and duplication events, respectively. Large databases providing orthologs exist [14–16]; however, existing approaches for ortholog detection are based on graph structures or gene trees and therefore rely on existing gene annotations (knowledge of gene locations in the assembly) [17–19]. Since gene annotation is challenging, the generation of high-quality genomes greatly surpasses that of accurate annotations, resulting in a growing genome annotation gap, that is, many available genomes lack gene annotations (Figure 2).

Several new groundbreaking methods address this shortage of annotations and orthology information. TOGA (Tool to infer Orthologs from Genome Alignments) leverages whole genome alignments of unannotated genomes to an annotated reference genome and a machine learning classifier to integrate genome annotation and orthology inference [20]. By scaling linearly with the number of unannotated genomes, TOGA can generate annotations and ortholog data sets for hundreds of species [10,20,21]. However, TOGA infers orthologous loci from alignments of neutrally evolving (intergenic and intronic) regions, which become randomized with increasing molecular distance. Accordingly, TOGA’s application range targets clades with partially aligning neutral regions, exemplified by birds or placental mammals [22]. Thus, graph- or gene tree–based methods are needed for orthology inference across larger evolutionary distances. While these methods have quadratic runtimes, preventing large-scale applications, new methods like fastOMA provide linear scalability, enabling orthology inference across hundreds of species [18]. Furthermore, deep learning–based ab initio gene predictors provide rapid and accurate annotations for diverse species [23–25]. With growing genomic training data, further increases in accuracy are expected.

### Progress in linking genome evolution to phenotypic evolution

Large-scale comparative genomics analyses need phylogenetic frameworks. Thus, expanding genomic resources calls for scalable tools to infer phylogenies from whole genomes. A recent phylogenomic analysis of birds showed that including more genomic loci increases phylogenetic resolution and that intergenic regions can avoid biases observed with gene sequences [26]. Building on these findings, CASTER, a new scalable discordance-aware approach, directly infers phylogenies from multiple genome alignments with efficient runtimes [27].

Several methods focus on repeated phenotypic evolution and screen for recurrent genomic divergence patterns to link phenotypes to genotypes. Many of these methods can be applied to diverse genomic elements, including exons of coding or noncoding genes or regulatory elements. Divergence patterns are either estimated as evolutionary rates, which considers only substitutions [28–31] or as sequence divergence, which also incorporates insertions and deletions and allows detecting losses of functional elements [32]. Phenotypic associations are detected by comparing the divergence of genomic elements across species with different phenotypic states. Recent advancements in tools like PhyloAcc, which models substitution rates across target lineages, increase the robustness of results by taking possible gene tree discordances into account [31] and advance our possibility to link substitution rate changes to continuous trait evolution [30].

Beyond convergent phenotypes, lineage-specific phenotypes can be analyzed by identifying genomic elements that exhibit exceptionally high divergence [33]. For example, given the substitution rate of neutrally evolving genomic regions, phyloP detects accelerated genomic elements that evolved faster than expected under neutrality [34]. Since species exhibiting the focal phenotype may also share other traits [35], integrating functional annotations helps to identify elements related to the focal phenotype (Figure 1).

While methods applicable to any class of genomic elements enable unbiased genomic screens, additional power to detect functionally relevant genomic changes can be gained by considering how specific mutations affect genes or regulatory elements. For coding genes, distinguishing between synonymous and nonsynonymous substitutions enables detecting signatures of selection that can indicate functional differences. Using annotated genomes and reliable orthology inferences, a variety of tools screen for intensified, relaxed, and positive selection [36–38]. These methods are rapidly developing to decrease runtimes [38] and increase accuracy by taking into account synonymous rate variation, multinucleotide codon substitutions, and alignment errors [39]. In addition to selection signatures, one can screen for gene losses associated with phenotypic differences [35,40].

Regulatory elements such as promoters and enhancers are key contributors to phenotypic evolution. To obtain aligning regulatory element candidates, one often uses conserved noncoding elements (GNEs) that evolve under evolutionary constraint. Phylogenetic methods such as phyloP identify CNEs from multiple genome alignments [41]. Detecting functionally relevant divergence in regulatory elements and associating them with phenotypic differences can be improved by quantifying divergence of transcription factor–binding sites (TFBS). For example, REforge extends the Forward Genomics framework to measuring TFBS instead of sequence divergence [42]. Deep learning–based methods like TACIT (Tissue-Aware Conservation Inference Toolkit) directly learn to predict tissue-specific enhancer activity from the DNA sequence, which increases the ability to associate candidate enhancers with phenotypes [43].

### Key insights gained from comparative genomics

Recent studies achieved breakthroughs in understanding genome evolution underpinning phenotypic differences. These studies span diverse biological systems to explore intriguing traits with biomedical relevance, such as cancer resistance, longevity, and viral tolerance, and enigmatic phenotypes related to cognition, metabolism, and body plan evolution.

**Figure 3:**
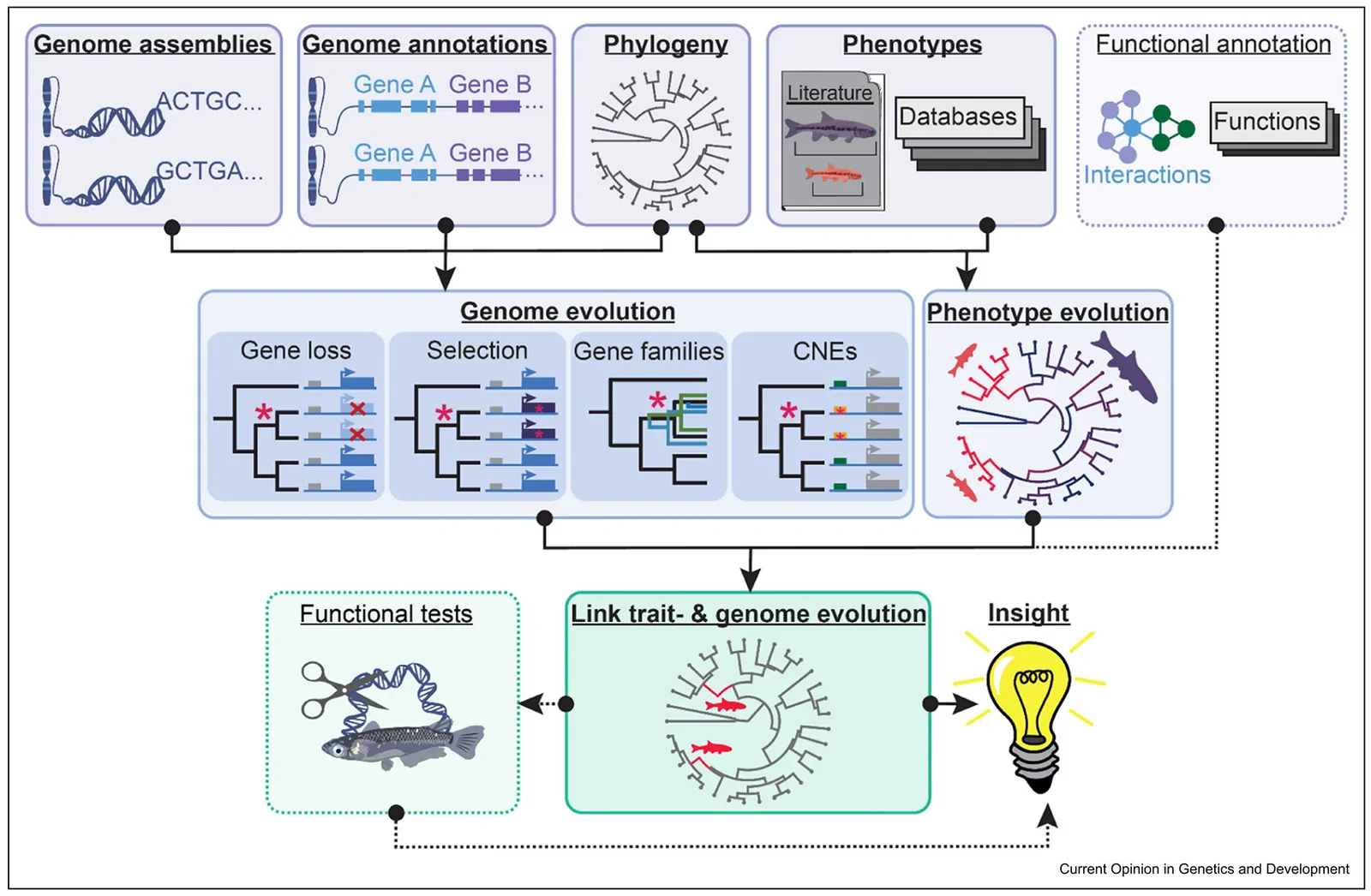
Highlight findings from studies linking genotypes to phenotypes. This figure illustrates recent discoveries regarding the molecular basis of enigmatic phenotypes and phenotypes of high biomedical interest. Icons illustrate some of the organismal diversity used by recent studies, the key molecular mechanisms, and whether studies employed functional tests to explore the effect of candidate loci.

Large-scale comparative genomics of long-lived and/or large-bodied species provided insights into the molecular basis of longevity and cancer resistance (Figure 3) [44–48]. For example, duplications of tumor suppressor genes, including *TP53* retrogene expansions, in elephants likely help to avoid cancer [48]. Furthermore, screening for positive selection signatures among protein-coding genes from > 250 mammals showed that genes positively selected in elephants were enriched in anticancer functions highlighting candidate genes that likely contribute to cancer resistance, such as the DNA damage repair genes *RIF1, NEK4*, and *KAT5* and the cell-death related genes *CASP8, APAF1*, and *BCLAP* [44]. Duplications of anticancer genes likely also promoted the evolution of large body sizes and longevity in whales as indicated by recent work on the genomes of the exceptionally long-lived bowhead whale [46] and the blue whale, the largest species that ever lived [45]. In the bowhead whale, cell culture experiments indicate that expression of a retroduplicated *CDKN2C* gene helps prevent DNA damage accumulation, providing a potential mechanism for its extended lifespan [46].

Beyond large-bodied species, many bats exhibit long lifespans relative to their small size. Long-lived *Myotis* bats exhibit signatures of positive selection in genes involved in cancer and double-strand DNA break repair and enhancer activity losses contributing to cancer resistance [49,50]. Similarly, analyses of genome evolution in rockfishes with varying lifespans indicated that positive selection on DNA maintenance and repair pathway genes (*WRAP53, DCLRE1B, FEN1*, and *MCM6*) and duplications in the butyrophilin gene family (*BTN* and *BTNL* genes) contribute to extended lifespans [47]. Together, these findings identify candidate genes, pathways, and mechanisms by which genome evolution shapes longevity and cancer resistance across diverse lineages.

Recent work has unveiled molecular mechanisms of bats’ exceptional disease resistance and viral tolerance (Figure 3) [21,51]. Using TOGA to identify 17 130 orthologs across 115 mammals and screening for signatures of selection, researchers detected an excess of immune gene adaptations in the stem-bat lineage and showed that signatures of positive selection on immune-related genes are more prevalent in bats compared to other mammalian orders. These genes included key factors for viral entry (e.g. *ANPEP, SCARB1*, and *CTSB*) and sensing (e.g. *TLR8*), and regulators of antiviral and inflammatory responses (e.g. *IFNB1, IL-17A*) that likely help prevent uncontrolled inflammation during viral infections. Cellular infection experiments further highlighted antiviral differences of *ISG15*, which evolved a strong anti-SARS-CoV-2 activity in the two bat families most frequently associated with coronaviruses [21]. Furthermore, a study focusing on horseshoe bats supported widespread positive selection on immune genes and discovered expansions of immune genes, such as *ANXA2R* [51]. Using cell culture experiments, the authors confirmed dosage-dependent inhibition of viral replication by *ANXA2R*, establishing a role of this gene expansion in enhanced antiviral defense.

Vocal learning evolved convergently in humans, whales, bats, and seals [52]. Researchers integrated vocalization-associated epigenomic data with comparative genomics across > 200 placental mammals. Analyzing TOGA-identified orthologs across 215 mammals identified limited convergence. In contrast, training the machine learning–based TACIT on open chromatin data from a brain region implicated in vocal production identified 50 candidate enhancers associated with vocal learning. Many of these exhibited reduced activity in vocal learners and were located near genes linked to speech impairments in humans [52], suggesting that convergent activity losses of motor cortex regulatory elements promoted the evolution of vocal learning (Figure 3).

In addition to enhancer activity losses, recent work highlighted coding gene losses as important drivers of phenotypic evolution. For example, adaptive gene loss likely played a crucial role in the evolution of hovering flight in hummingbirds [53]. Hummingbirds lost the gluconeogenic muscle enzyme *FBP2* around a time period during which hovering flight evolved. Knockdown of *FBP2* in bird myoblasts increased glycolysis and mitochondrial respiration, suggesting that *FBP2* loss contributed to metabolic muscle adaptations required for energy-demanding hovering flight [53].

Penguins are flightless birds possessing many adaptations to survive in extreme environments. Large-scale comparative genomics identified positive selection, gene family expansions, and losses of genes related to dietary adaptations, vision, oxygen transport, diving, and thermoregulation, thus revealing genomic changes involved in the transition to a secondary aquatic lifestyle and Arctic adaptation in penguins [54],

In moles, females exhibit both ovaries and androgen-producing testes (ovotestes), which result in masculinized external genitalia, large muscle mass, and aggressive behavior. Comparative genomics uncovered that duplication of enhancers controlling expression of the androgen synthesis gene *CYP17A1* and genomic rearrangements that alter enhancer–promoter interactions in the *FGF9* locus contribute to these traits [55].

Limbs exhibit high morphological diversity among vertebrates with numerous convergent changes. For example, several bird lineages, including penguins, kingfishers, and swallows, independently evolved short legs [56], Comparative genomics revealed convergent evolutionary rate acceleration of CNEs located near limb developmental genes in these birds, whereas coding genes did not exhibit signatures of convergence [56]. Similarly, accelerated evolution of different enhancers of *EMX2* promoted the convergent evolution of marsupial gliding membranes [57].

Additionally, comparative genomics implicated sequence and TFBS divergence in many limb regulatory elements in the convergent loss of limbs in snakes and other reptiles [58,59]. Snakes (but not other limbless reptiles) also lost two limb developmental genes *HOXD12* and *TWIST2* [58], and microdeletions observed in snake *PTCH1* inhibit digit growth when introduced in a mouse model [59]. Divergence in CNEs near genes implicated in jaw morphogenesis, somite size, and lung development further reveals potential regulatory changes contributing to unique skull morphology, body elongation, and asymmetric lungs in snakes [59]. Overall, these studies indicate that changes in regulatory elements that control expression of pleiotropic developmental genes are major drivers of morphological evolution.

### Collective insights from recent large-scale comparative genomics studies

Collectively, these studies highlight the power of comparative genomics in discovering genomic candidates contributing to phenotypic evolution, which can be tested in cellular or model organism systems [21,46,49–51,53,59,60], Studies that investigate several types of genomic changes often reveal that multiple molecular mechanisms affecting genes (duplication, loss, positive selection) and gene regulation (driven by CNE or enhancer evolution) collectively contribute to phenotypic change. Among these mechanisms, subtractive changes like enhancer activity losses [5,50,52,61] and gene losses [8,53,54,59,60] may be underappreciated drivers of adaptive phenotypic changes. This could stem from a cognitive bias in humans favoring additive solutions over subtractive ones [62]. Despite important exceptions, these studies also support a higher relative importance of gene regulation in the evolution of skeletal morphology or body plans, while both coding and regulatory evolution contribute to metabolic or physiological differences.

### Outlook and conclusions

Advances in sequencing, annotation, and analytical tools have propelled comparative genomics into a new era, enabling unprecedented insights into the molecular basis of phenotypic diversity. Studies leveraging these resources highlight the interplay of coding and noncoding changes, gene duplications, and losses in shaping the phenotype. However, challenges remain, including the need for more efficient comprehensive genome annotations. Future progress will depend on refining analytical methods, integrating phylogenetic methods, and bridging comparative genomics with functional validation to fully unravel the genetic basis of evolutionary change.

## Data Availability

No data were used for the research described in the article.

## Box 1 Current challenges and limitations.

1. Lineage-specific molecular evolution
  Assessing the contributions of lineage-specific molecular evolution to a focal phenotype remains challenging. Convergent phenotypes are often used as ‘evolutionary replicates’ to identify associations with repeated genomic changes, implicitly assuming that the same loci underlie convergent phenotypic evolution. While this is a powerful approach, exclusively searching for signals of genetic repeatability in the *same* elements misses the contribution of lineage-specific changes in *different* elements to convergent phenotypes, and overestimates the degree of convergent molecular evolution. However, genomic changes, including selection, acceleration, and loss signatures, can be mapped to single lineages or clades, enabling the identification of lineage-specific changes. Integrating existing functional annotations of genes or regulatory elements allows to identify lineage-specific changes that likely contribute to a focal trait. While the effectiveness of this approach depends on how complete the annotations are, it offers more comprehensive insight into the contribution of repeated and lineage-specific genomic changes.
2. Experimental validation
  Not all molecular changes associated with a certain phenotype are contributing to that phenotype. Thus, experiments are fundamental to turn associations into functional insights. Since comparative genomics frequently identifies numerous testable candidates, this creates a validation bottleneck. Consequently, investigators often prioritize candidates with known functions over uncharacterized elements, missing opportunities to leverage genomic insights for targeted experiments. For example, a genome-wide screen for inactivated genes in mammals with poor vision predicted novel eye-related genes [60]. Using a zebrafish knockout confirmed a role of the uncharacterized *SERPINE3* in vertebrate eyes [60]. Thus, increasing experimental throughput will be crucial to test more candidates, including uncharacterized genes. While whole-organism studies remain essential for developmental or organism-level phenotypes [55], cellular systems and organoids enhance feasibility and throughput for investigating metabolic, cellular, and organ-level phenotypes [63,64]. Fortunately, advancements in generating pluripotent stem cells and organoids from various species [64] as well as single-cell RNA- and ATAC-seq and massively parallel reporter assays [33,61,65] provide powerful new systems offering increased throughput for experimental validation.
  Another challenge is to appropriately recapitulate naturally occurring genomic differences in experimental systems. For example, while gene losses can be recapitulated by gene knockout or knockdown [53,59,60], amino acid changes may enhance, diminish, or alter gene functions. Duplicated genes may simply increase expression levels, which can be mimicked by overexpression, but this fails to detect possible neofunctionalization of one gene copy. Additionally, mutations in CNEs may have unpredictable effects depending on whether the regulatory element acts as an enhancer or silencer [66] and whether mutations disrupt or create activating or repressing TFBS [61] . CRISPR-Cas9 provides a powerful tool to faithfully reproduce naturally occurring genetic changes in experimental models.
3. Comprehensive, computer-accessible phenotypes
  With sequenced genomes available for thousands of species, a key challenge in associating phenotypic to genomic differences has shifted to the availability of systematic knowledge of phenotypic traits. Phenotypes are often quantitative and complex, that is, they may vary gradually and comprise multiple morphological, physiological, and life-history adaptations, making them more difficult to describe and compare than DNA. Even presence–absence traits can be difficult to classify, exemplified by the highly reduced but present clavicle in rabbits. Furthermore, phenotypic knowledge of curators or contained in monographs is often not available in a standardized and computer-accessible form. Filling this gap by digitization and new phenotyping is essential for linking phenotype to genotype [67].
4. Accurate, scalable, and accessible analytical tools
  While substantial progress has been made in developing accurate and scalable comparative genomics methods, further improvements will be necessary. Accurately annotating all coding or noncoding genes, though deep learning methods fed with massive high-quality training data offer great promises [68]. Selection results obtained with different methods or data sets often differ [10,69], calling for ways to increase robustness [39]. Aligning whole genomes or even coding sequences is not trivial, and recent selection methods already take alignment errors into account [39]. Finally, developing easy-to-use pipelines to integrate new methods and automatize analyses after genome assembly [70], including genome alignment, phylogenomics, orthology inference, gene alignment, and selection screens will help accessibility of recent methods and further promote progress in the field.
